# Effect of the Melanocortin 4-Receptor Ile269Asn Mutation on Weight Loss Response to Dietary, Phentermine and Bariatric Surgery Interventions

**DOI:** 10.3390/genes13122267

**Published:** 2022-12-01

**Authors:** Itzel G. Salazar-Valencia, Hugo Villamil-Ramírez, Francisco Barajas-Olmos, Martha Guevara-Cruz, Luis R. Macias-Kauffer, Humberto García-Ortiz, Omar Hernández-Vergara, David Alberto Díaz de Sandy-Galán, Paola León-Mimila, Federico Centeno-Cruz, Luis E. González-Salazar, Rocío Guizar-Heredia, Edgar Pichardo-Ontiveros, Leonor Jacobo-Albavera, Rosalinda Posadas-Sánchez, Gilberto Vargas-Alarcón, Rafael Velazquez-Cruz, Ruth Gutiérrez-Aguilar, Carlos Zerrweck, Héctor Isaac Rocha-González, Juan Gerardo Reyes-García, Miriam del C. Carrasco-Portugal, Francisco Javier Flores-Murrieta, Armando R. Tovar, Lorena Orozco, Teresa Villarreal-Molina, Samuel Canizales-Quinteros

**Affiliations:** 1 Unidad de Genómica de Poblaciones Aplicada a la Salud, Facultad de Química, Universidad Nacional Autónoma de México (UNAM), Mexico City 14610, Mexico; 2 Instituto Nacional de Medicina Genómica (INMEGEN), Mexico City 14610, Mexico; 3 Programa de Maestría en Ciencias Bioquímicas, Facultad de Química, Universidad Nacional Autónoma de México (UNAM), Mexico City 04510, Mexico; 4 Laboratorio de Immunogenómica y Enfermedades Metabólicas, Instituto Nacional de Medicina Genómica (INMEGEN), Mexico City 14610, Mexico; 5 Departmento de Fisiología de la Nutrición, Instituto Nacional de Ciencias Médicas y Nutrición Salvador Zubirán, Mexico City 14080, Mexico; 6 Laboratorio de Genómica de Enfermedades Cardiovasculares, Instituto Nacional de Medicina Genómica (INMEGEN), Mexico City 14610, Mexico; 7 Departamento de Endocrinología, Instituto Nacional de Cardiología Ignacio Chávez (INCICh), Mexico City 14080, Mexico; 8 Departamento de Biología Molecular, Instituto Nacional de Cardiología Ignacio Chávez (INCICh), Mexico City 14080, Mexico; 9 Laboratorio de Genómica del Metabolismo Óseo, Instituto Nacional de Medicina Genómica (INMEGEN), Mexico City 14610, Mexico; 10 Laboratorio de Enfermedades Metabólicas: Obesidad y Diabetes, Facultad de Medicina, Universidad Nacional Autónoma de México (UNAM), Hospital Infantil de México “Federico Gómez”, Mexico City 06720, Mexico; 11 Clínica de Obesidad del Hospital General Tláhuac, Mexico City 13250, Mexico; 12 Facultad de Medicina, Alta Especialidad en Cirugía Bariátrica, Universidad Nacional Autónoma de México (UNAM), Mexico City 04510, Mexico; 13 Sección de Estudios de Posgrado e Investigación, Escuela Superior de Medicina, Instituto Politécnico Nacional, Mexico City 11340, Mexico; 14 Unidad de Investigación en Farmacología, Instituto Nacional de Enfermedades Respiratorias Ismael Cosío Villegas, Mexico City 14080, Mexico

**Keywords:** melanocortin-4-receptor, Ile269Asn-mutation, obesity, weight-loss, interventions

## Abstract

The loss of function melanocortin 4-receptor (*MC4R*) Ile269Asn mutation has been proposed as one of the most important genetic contributors to obesity in the Mexican population. However, whether patients bearing this mutation respond differently to weight loss treatments is unknown. We tested the association of this mutation with obesity in 1683 Mexican adults, and compared the response of mutation carriers and non-carriers to three different weight loss interventions: dietary restriction intervention, phentermine 30 mg/day treatment, and Roux-en-Y gastric bypass (RYGB) surgery. The Ile269Asn mutation was associated with obesity [OR = 3.8, 95% CI (1.5–9.7), *p* = 0.005]. Regarding interventions, in the dietary restriction group only two patients were *MC4R* Ile269Asn mutation carriers. After 1 month of treatment, both mutation carriers lost weight: −4.0 kg (−2.9%) in patient 1, and −1.8 kg (−1.5%) in patient 2; similar to the mean weight loss observed in six non-carrier subjects (−2.9 kg; −2.8%). Phentermine treatment produced similar weight loss in six carriers (−12.7 kg; 15.5%) and 18 non-carriers (−11.3 kg; 13.6%) after 6 months of pharmacological treatment. RYGB also caused similar weight loss in seven carriers (29.9%) and 24 non-carriers (27.8%), 6 months after surgery. Our findings suggest that while the presence of a single *MC4R* loss of function Ile269Asn allele significantly increases obesity risk, the presence of at least one functional *MC4R* allele seems sufficient to allow short-term weight loss in response to dietary restriction, phentermine and RYGB. Thus, these three different interventions may be useful for the short-term treatment of obesity in *MC4R* Ile269Asn mutation carriers.

## 1. Introduction

Approximately 5% of severe obesity cases are associated with loss of function mutations in the hypothalamic appetite-regulating melanocortin-4 receptor (*MC4R*), the most common cause of monogenic non-syndromic obesity [1,2]. Ile269Asn has been identified as a loss of function *MC4R* mutation linked to morbid obesity and type 2 diabetes (T2D) in Latinos [3,4], by impairing both cAMP production and MC4R internalization by β-arrestin [5,6,7]. According to the Genome Aggregation Database (gnomAD), the minor allele frequency of this mutation is 1% in Latinos. Notably, a study of 640,000 multiethnic exomes found the Ile269Asn mutation present only in the Mexican population (minor allele frequency, 1%) and was associated with obesity [8]. Independent studies in this population previously reported this association in both children and adults [9].

There is currently no standardized protocol for obesity treatment in patients with *MC4R* mutations. Some studies have analyzed the ability of patients with *MC4R* mutations to lose weight with lifestyle changes, pharmacological treatment, and bariatric surgery. Studies assessing the response to lifestyle changes have reported inconsistent results. On one hand, pediatric patients with obesity and *MC4R* mutations other than Ile269Asn showed weight loss in response to a controlled and intensive program of restricted dieting and daily physical activity [10], while weight loss was not achieved in an independent study of European children and adolescent *MC4R* mutation carriers in response to a less intensive dietary program, suggesting the need for personalized treatment based on *MC4R* genotype [11]. Multiple studies have reported different weight loss success rates in response to bariatric surgery, showing considerable inter-individual variation [12,13]. Most studies analyzing the response to Roux-en-Y gastric bypass (RYGB) report similar short-term weight loss in patients with and without *MC4R* mutations [14,15], although long-term results showed weight gain in patients with certain *MC4R* variants [16]. Regarding pharmacological treatment, one of the first reports comparing weight loss according to *MC4R* genotype was conducted in a reduced number of European patients, reporting similar weight loss in patients with and without *MC4R* mutations treated with the MC4R agonist setmelanotide [3]. Moreover, the GLP-1 receptor agonist liraglutide was found to induce similar weight loss in obese patients with and without *MC4R* mutations, suggesting *MC4R* mutation carriers can be treated with this agonist [17]. Other widely used weight loss drugs such as phentermine have not been tested in individuals with *MC4R* mutations.

Because of the relatively high frequency of the Ile269Asn mutation in Mexicans with obesity, the purpose of the present study was to establish whether the response to dietary, phentermine and/or bariatric surgery interventions in Mexican patients with obesity is affected by the presence of this mutation.

## 2. Materials and Methods

### 2.1. Study Populations

#### 2.1.1. Case and Control Association

**Figure 1:**
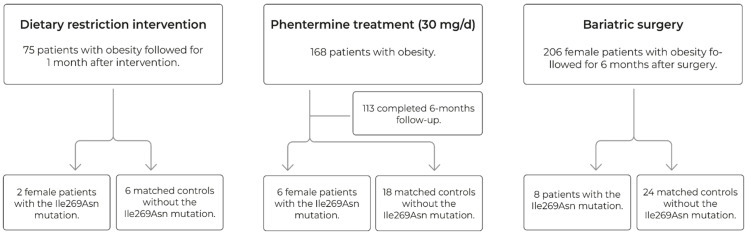
Flow diagram: an overview of Ile269Asn carrier and matched non-carrier selection.

A total of 1683 adults aged 18–81 years (483 normal-weight controls and 1200 obesity cases) were genotyped to assess the association of *MC4R* Ile269Asn with obesity. Control individuals mainly belonged to the GEA cohort [18]. Recruitment and inclusion criteria have been previously described, and all selected normal-weight controls were aged > 40 years [18,19]. The obesity group included 75 individuals submitted to dietary intervention, 168 treated with phentermine, and 206 submitted to bariatric surgery. These weight loss interventions were compared in individuals with and without the *MC4R* Ile269Asn mutation, as described in the flow diagram (Figure 1).

#### 2.1.2. Dietary Intervention

A cohort of 75 Mexican mestizo adults aged 18–60 years with BMI ≥ 30 kg/m^2^ were selected for dietary intervention. Inclusion and exclusion criteria were previously published [20]. Briefly, participants received menus and a thirty-day prescription with dietary indications of 750 kcal/d energy restriction based on their habitual total energy expenditure, as previously described [21]. This diet was designed with the following macronutrient distribution: 50% energy content from carbohydrates, 20–25% from protein and 25–30% from fat. Patients received a weekly food supply with 80% of the foods contained in the menu to improve compliance. Participants were advised to maintain their usual level of physical activity during the month of dietary intervention.

#### 2.1.3. Phentermine Intervention

In this prospective, phase IV open-label study, 168 volunteers aged above 18 years and with a BMI ≥ 30 kg/m^2^ were recruited. The purpose of the study was to evaluate the efficacy and safety of oral administration of 30 mg of phentermine in obese patients after 6 months of treatment. Inclusion and exclusion criteria were previously described [22]. Patients received medical support and were instructed to follow a 1500 Kcal/d diet (50% energy content from carbohydrates, 30% from fat and 20% from protein) and to perform 20 min/d physical activity.

#### 2.1.4. Bariatric Surgery

A total of 206 female patients undergoing RYGB were recruited from the Obesity Clinic at the Hospital General de Tlahuac in Mexico City. Interventions included laparoscopic RYGB performed by three surgeons using the same operative techniques; the surgical methods have been described previously [23]. After surgery, moderate effort exercise (250–400 min per week) and moderate-to-high resistance exercise (12–15 repetitions for 3 series/large muscle group) were recommended.

#### 2.1.5. Ile269Asn Carrier and Non-Carrier Matching in the Three Weight-Loss Intervention Groups

Age and BMI matching between mutation carriers and non-carriers (all female) in each intervention group was performed by selecting patients within similar baseline age and BMI ranges, seeking the closest match available. Three non-mutation carrier controls were selected for each mutation carrier. The highest difference between matched carriers and non-carriers was 7 years of age and 3 kg/m^2^ BMI in the dietary intervention group, 5 years of age and 3 kg/m^2^ BMI in the phentermine intervention group, and 5 years of age and 7 kg/m^2^ BMI in the bariatric surgery group.

### 2.2. Anthropometric Measurements

Height and weight were measured following standard protocols with calibrated instruments as previously described [18,19]. BMI was calculated as body weight in kilograms divided by the square of height in meters (kg/m^2^). Obesity was defined as a BMI ≥ 30 kg/m^2^, class I/II obesity as 30 kg/m^2^ ≤ BMI < 40 kg/m^2^, and class III obesity as BMI ≥ 40 kg/m^2^. Normal weight was defined as BMI < 25 kg/m^2^ and ≥18.5 kg/m^2^ according to World Health Organization (WHO) criteria [24]. All measurements were recorded before and after the dietary, pharmacologic, or bariatric surgery intervention. Weight loss percentage (%WL) was calculated as (initial weight minus post-treatment)/(initial weight) × 100. The percentage of excess weight loss (%EWL) was calculated as (preoperative body weight minus follow-up body weight)/(preoperative body weight minus ideal body weight) × 100. Skeletal muscle mass and fat mass were measured using Inbody 720 multifrequency bioimpedance analysis (Biospace, Co. Cerritos, CA, USA) in the patients submitted to dietary intervention, and with an electric bioimpedance instrument (Omron HBF-514C) in those treated with phentermine.

### 2.3. Biochemical Measurements

Blood samples were drawn after 8–12 h of overnight fasting to determine serum levels of glucose by enzymatic assays. Serum insulin, leptin and adiponectin levels were measured by ELISA in the dietary intervention group, and by BIO-RAD BIO-PLEX Luminex System in the phentermine group. Insulin resistance was estimated using the homeostasis model assessment of insulin resistance (HOMA-IR) [25]. T2D was defined by a prior diagnosis, use of glucose-lowering medications, and/or fasting serum glucose levels ≥ 126 mg/dL [26]. All measurements were performed before the intervention and at different follow-up times.

### 2.4. MC4R Ile269Asn Genotyping

Genomic DNA was isolated from peripheral leukocytes using standard methods. *MC4R* Ile269Asn genotypes were obtained from the Multi-Ethnic Genotyping Array (MEGA, Illumina, San Diego, CA, USA) for all participants except those in the dietary intervention group, where samples were genotyped using Taqman probes. The variant did not deviate from Hardy–Weinberg equilibrium in any group. Global ancestry was estimated as previously described [19].

### 2.5. Local Ancestry Inference

Local ancestry along chromosome 18 was estimated in the 206 patients from the bariatric surgery group, using 50 European and 50 African individuals from the 1000 Genomes project (Phase 3) and 50 Native Americans from the MAIS cohort as reference populations [27]. Phasing was performed using SHAPEIT v2.17. Local ancestry estimation was performed using RFMix v2 with two EM iterations and a forward–backward threshold of 0.9 [28]. The mean Native American ancestry proportion of a 1Mb segment containing the *MC4R* Ile269Asn mutation was estimated in individuals with and without the mutation.

### 2.6. Statistical Analysis

Continuous variables are presented as mean ± standard error, and dichotomous variables as frequencies and percentages. Association of the Ile269Asn mutation with obesity was tested using logistic regression. This association was adjusted for sex and admixture including samples with available global ancestry estimations (482 controls and 1062 cases). The distribution of quantitative variables was evaluated using the Kolmogorov–Smirnov test; variables with non-normal distribution were log-transformed. Treatment effects between Ile269Asn mutation carriers and non-carriers were calculated using paired Student t test. Differences in the treatment effect between the groups were calculated using linear regression models. Statistical analyses were performed with the SPSS software package version 15.0 (SPSS, Chicago, IL, USA).

## 3. Results

### 3.1. Case–Control Association Study

In the case–control association study, 5/483 normal weight individuals (1%) and 45/1200 individuals with obesity (3.8%) were heterozygous for the *MC4R* Ile269Asn mutation. Only one homozygous individual who had class III obesity was found. Table 1 shows the association of the Ile269Asn mutation with overall obesity, and stratified according to obesity class. This mutation was significantly associated with increased overall risk of obesity (*p* = 0.005). Because the sex ratio and mean Native American ancestry proportion were significantly different in cases and controls (Supplementary Table S1), the association was adjusted for sex and admixture remaining statistically significant (*p* = 0.021). Although Ile269Asn genotypes were most frequent in the obesity class III group (5.5%), the variant was significantly associated with all obesity classes (*p* < 0.05).

Mean Native American ancestry of the chromosome 18 segment containing the *MC4R* Ile269Asn mutation was estimated in 206 patients with obesity, and was 58% in wildtype homozygous individuals, 79% in heterozygous patients and 100% in the Asn269Asn homozygous patient (Supplementary Table S2).

### 3.2. Response to Dietary Intervention

In the dietary intervention group (*n* = 75), only two women carried the *MC4R* Ile269Asn mutation (patient 1: basal BMI of 45.2 kg/m^2^ and patient 2: basal BMI of 46.4 kg/m^2^ with T2D). Six age and sex matched non-carriers with BMI > 40 kg/m^2^ were used as controls for comparisons (Table 2). After 1 month of dietary intervention, both *MC4R* mutation carriers lost weight: −4.0 kg (−2.9%) in patient 1, and −1.8 kg (−1.5%) in patient 2; while mean weight loss in the control group was −2.9 ± 0.6 kg (−2.8 ± 0.6%). Weight loss was accompanied by a decrease in fat mass and serum leptin levels in both mutation carriers and controls. Serum glucose levels increased after 1 month of dietary intervention in patient 1, from 99.5 to 105.3 mg/dL, but decreased in patient 2, from 139.6 to 117.9 mg/dL, and in controls (mean −7.6 ± 4.8 mg/dL).

### 3.3. Response to Phentermine Treatment after Six Months

Overall, 113/168 patients who received phentermine treatment completed the 6 month follow-up. Six of the participants (4%, all women) carried the *MC4R* Ile269Asn mutation. A total of 18 age-, gender-, and initial BMI-matched individuals without the Ile269Asn mutation were included as controls.

**Figure 2:**
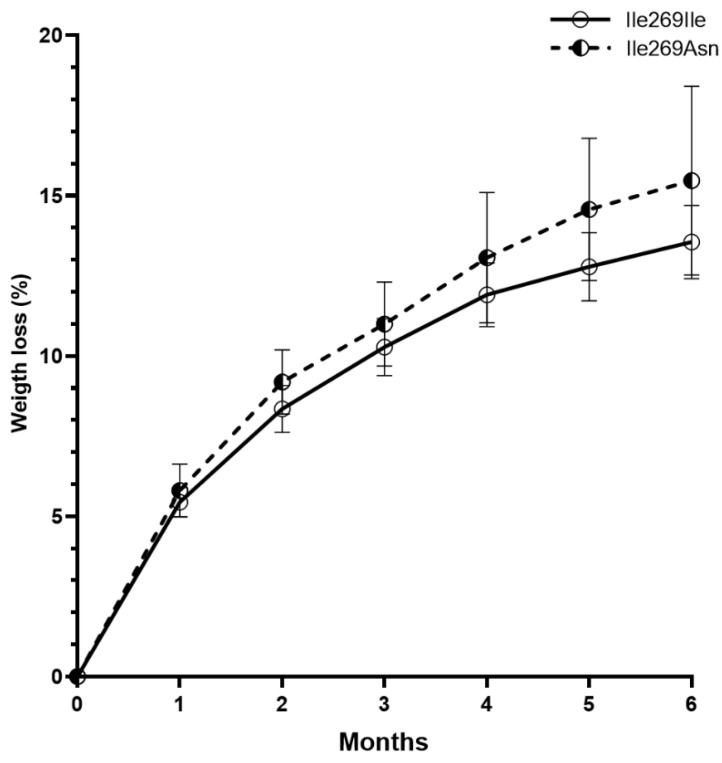
Six months of phentermine treatment led to weight loss in Ile269Asn carriers (*n* = 6) and non-carriers (*n* = 18). Data are presented as mean ± SEM.

After 6 months of phentermine treatment, weight was significantly reduced in both groups (−12.7 ± 2.3 kg, *p* = 0.003 in *MC4R* mutation carriers and −11.3 ± 0.9 kg, *p* < 0.001 in non-carriers), with no significant difference between carriers and non-carriers (*p* = 0.523; Table 3). Weight loss percentage was also similar in both groups (15.5 ± 2.9% in carriers and 13.5 ± 1.1% in non-carriers, *p* = 0.250) (Figure 2). Moreover, fat mass percentage decline was very similar in both groups, although the difference between fat mass percentage before and 6 months after phentermine treatment was significant only in non-carriers (*p* < 0.001). Systolic blood pressure decreased significantly after treatment only in non-carriers (*p* = 0.027), with no significant difference between carriers and non-carriers (*p* = 0.100; Table 3).

Regarding glucose metabolism parameters, serum levels of glucose, insulin and HOMA-IR decreased in both *MC4R* mutation carriers and controls, without significant differences between groups. Notably, in *MC4R* Ile269Asn mutation carriers, serum leptin levels showed a 4-fold decline after 3 months (basal 24.7 ± 6.9 ng/mL and 6.3 ± 1.6 ng/mL after 3 months, *p* = 0.018) and a modest increase after 6 months of phentermine treatment, without reaching pre-treatment levels. In contrast, in non-carriers, serum leptin levels showed a moderate decline at 3 months (basal 20.2 ± 1.8 ng/mL and 18.9 ± 3.2 ng/mL after 3 months; *p* = 0.146), which continued to decline after 6 months (15.2 ± 3.3 ng/mL; *p* = 0.039). Moreover, differences in leptin levels between groups were significant after 3 months (*p* = 0.012, Supplementary Table S3), but not after 6 months of phentermine treatment (*p* = 0.484; Table 3).

The most frequent phentermine-related adverse events were categorized as gastrointestinal, neurological or psychiatric. All adverse events were reported as of mild or moderate intensity in both Ile269Asn mutation carriers and non-carriers (Supplementary Table S4).

### 3.4. Response to RYGB Surgery

**Figure 3:**
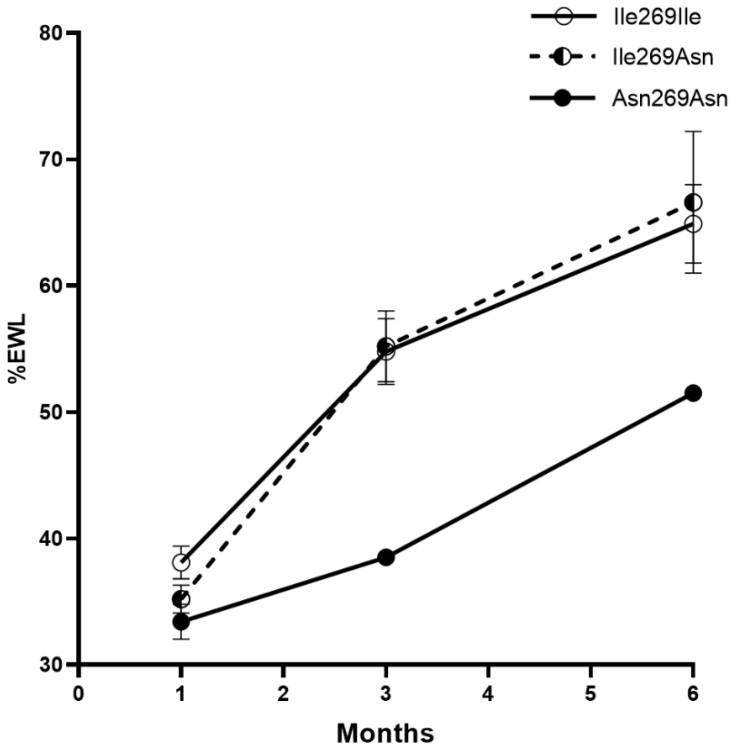
Percentage of excess weight loss (%EWL) was similar in heterozygous carriers (*n* = 7) and non-carriers (*n* = 24) 6 months after RYGB surgery. %EWL was lower in the Asn269Asn homozygous patient (*n* = 1). Data are presented as mean ± SEM.

To determine whether the response to bariatric surgery differs according to the presence of the *MC4R* Ile269Asn mutation, we genotyped 206 female patients with obesity previously submitted to RYGB. Of these, seven (3.4%) were heterozygous and one was homozygous. Twenty-four age-, sex- and initial BMI-matched non-carriers were used as controls. Six months after the RYGB, %WL and %EWL were similar in carriers (29.9% and 66.6%, respectively) and non-carriers (27.8% and 64.9%, respectively; Table 4), and the %EWL pattern over time was similar in both groups (Figure 3). Notably, the Asn269Asn homozygous patient had one of the highest baseline weights and BMIs (138 kg and 51.6 kg/m^2^, respectively), but showed a %WL similar to that of the heterozygous and non-carrier patients. However, the homozygous patient showed the lowest %EWL over time (51.5% after 6 months; Figure 3), likely because of the higher baseline weight. Hb1Ac levels did not differ significantly before or after RYGB in carriers and non-carriers.

## 4. Discussion

As previously reported in other Mexican cohorts, the *MC4R* Ile269Asn mutation was associated with adult obesity in the present study. This mutation was formerly associated with childhood and adult obesity in Mexico [8,9], and in vitro studies showed that it results in complete loss of function of the MC4R protein [7]. Although the Ile269Asn mutation is extremely rare or absent in non-Latino populations [8], mutation carrier frequency was 3.4 and 5.4% in Mexicans with class I/II and III obesity, respectively. This mutation has been proposed as the most important genetic contributor to obesity in the Mexican population [9]. It has been suggested that the *MC4R* Ile269Asn mutation may have resulted from a founder event in the Native American population. In this regard, local ancestry analyses revealed that the percentage of Native American ancestry of the chromosomal segment containing Ile269Asn was higher in individuals bearing the *MC4R* Ile269Asn mutation than in non-carriers. Our observation is in agreement with the previous suggestion that Ile269Asn is a Native American founder mutation [9].

The loss of MC4R function may affect the response to dietary intervention through appetite regulation [11,29]. However in the present study, *MC4R* mutation carriers and controls showed similar weight loss, consistent with previous studies in patients with obesity and heterozygous *MC4R* mutations submitted to a controlled and intensive program of restricted dieting and daily physical activity for 6 weeks [10]. Together, these findings suggest that the presence of at least one functional *MC4R* allele is enough to achieve short-term weight loss during a restriction diet.

The present study provides evidence that Ile269Asn carriers with obesity can be successfully treated with phentermine. Specifically, 3 months of phentermine treatment (30 mg) resulted in clinically relevant weight loss, which was similar in Ile269Asn carriers (10.9 ± 3.2%) and non-carriers (8.5 ± 4.3%). Indeed, phentermine treatment led to greater weight loss than that reported with other pharmacological treatments such as liraglutide (5.7 ± 1.4% after 4 months) in patients with *MC4R* mutations. However it must be considered that the latter cohort did not receive lifestyle counseling, diet or exercise interventions [17]. Additional studies are required to assess whether phentermine treatment induces effective weight loss in subjects with other *MC4R* mutations.

In addition to weight loss, patients showed decreased fat mass, lower fasting serum glucose levels and improved HOMA-IR measurements in both groups (Ile269Asn carriers and non-carriers). This is important considering that this mutation has been associated with an increased risk of type 2 diabetes in the Latino population [30,31]. Moreover, decreased plasma leptin and increased adiponectin levels may be explained by the weight loss and fat percentage decreases observed in phentermine-treated patients. Notably, the decrease in plasma leptin levels was of significantly higher magnitude in Ile269Asn carriers 3 months after the intervention. However, it was transient, as no differences between carriers and non-carriers were observed 6 months after treatment. Further studies are required to assess whether this is mediated by an effect of phentermine on individuals with impaired MC4R signaling.

The mechanism of action of phentermine is not fully understood; however, it is known that phentermine induces norepinephrine (NE) and dopamine release by inhibiting its recapture in the hypothalamus [32,33]. In the rat model, NE injection into the hypothalamus was found to reduce food consumption, while NE depletion caused by ascending ventral NE axon lesions led to overeating, thus suggesting that NE promotes satiety [34,35]. However, other studies suggest an opposite effect of exogenous NE in the hypothalamus, decreasing satiety in rats [36,37]. These effects could be modulated by the energy homeostasis-regulatory proopiomelanocortin [38,39,40], which in turn regulates MC4R, promoting satiety [41,42,43]. Our finding of similar weight loss in heterozygous carriers and non-carriers suggests that weight loss can be achieved by phentermine treatment in the presence of at least one functional *MC4R* allele. Conversely, alternative MC4R signaling mechanisms may be involved in the phentermine-induced weight loss effect, such as dopamine signaling in the pre-frontal cortex known to inhibit the appetite.

Several studies have reported that weight loss after RYGB is similar in *MC4R* mutation carriers and non-carriers [14,15]. In accordance, weight loss response after RYGB was similar in heterozygous Ile269Asn and control patients. Notably, in a heterozygous *MC4R* murine knockout model, although energy balance was disrupted, causing obesity, these mice responded well to RYGB surgery, suggesting that the presence of a single functional *MC4R* copy does not impair RYGB-induced weight loss [15]. Altogether, this suggests that heterozygous *MC4R* Ile269Asn patients are appropriate candidates for RYGB. A small number of studies assessing the effect of bariatric surgery in homozygous *MC4R* mutation patients have been published, with inconsistent results for different mutations [44]. In the present study, the only Asn269Asn homozygous patient showed a %WL similar to that of heterozygous and non-carrier patients, but the lowest %EWL after 6 months, most likely due to her higher baseline weight. This suggests that Asn269Asn homozygosity does not impair RYGB-induced weight loss at least in the short term. It must be considered that despite the key role of *MC4R* in obesity, single mutations in *MC4R* might not be sufficient to significantly impact weight loss outcomes because of the contribution of other variants involved in polygenic obesity [45]. Some of these variants may contribute to obesity through other mechanisms implicated in body weight loss, requiring further study.

Some study limitations must be acknowledged. Firstly, only a small number of patients with the Ile269An mutation were included in each intervention group (*n* = 2 for diet, 6 for phentermine and 7 for bariatric surgery), and only one homozygous *MC4R* Asn269Asn carrier was identified in the bariatric surgery group. A larger number of heterozygous and homozygous patients are required to obtain adequate statistical power to confirm our findings. Moreover, it would be important to test a dosage effect in different intervention groups; however, homozygous patients are very rare, even in the Mexican population (0.0001%) [8]. In addition, we cannot rule out the presence of other mutations in the *MC4R* gene in study participants, because we did not sequence this gene. The phentermine group had a high dropout rate (32.8%). However, this rate is consistent with that of other pharmacotherapy trials for obesity [46,47]. Unfortunately, in the intervention groups, only female Ile269Asn patients were found. Thus, studies including male carriers are necessary because of the known sex differences in obesity and weight loss [48]. Finally, our follow-up after interventions was short (1 to 6 months), and because long-term studies have reported that patients with other *MC4R* mutations gain weight in the long term, longer follow-up studies are necessary for Ile269Asn carriers.

## 5. Conclusions

In conclusion, this is the first study to provide evidence that dietary restriction, pharmacological treatment with phentermine and bariatric surgery induced significant weight loss in Mexican patients with obesity in the short-term, even in those bearing the Ile269Asn mutation. Thus, these three different interventions may be useful for the short-term treatment of obesity in *MC4R* Ile269Asn mutation carriers.

## Figures and Tables

**Table 1:** Association study of the *MC4R* Ile269Asn mutation with obesity.

Stratified on BMI Level	*n*	Genotype *n* (%)		OR (95% CI)	*p*-Value
		Ile269Ile	Ile269Asn/Asn269Asn		
Normal weight	483	478 (99.0)	5/0 (1.0)		
OB I–III	1200	1154 (96.2)	45/1 (3.8)	3.8 (1.5–9.6)	0.005
OB I/II	942	910 (96.6)	32/0 (3.4)	3.4 (1.3–8.7)	0.012
OB III	258	244 (94.6)	13/1 (5.4)	5.5 (1.9–15.4)	0.001

Data are *n* (%). *p*-values and ORs were calculated by logistic regression analysis. OR, odds ratio; CI, confidence interval. OB I–III, whole obesity group; OB I, class I obesity; OB II, class II obesity, and OB III, class III obesity.

**Table 2:** Comparison of anthropometric and biochemical parameters after 1 month of dietary energy restriction in Ile269Asn carriers and non-carriers.

	Ile269Asn *MC4R* Patient 1			Ile269Asn *MC4R* Patient 2			Control Group *n* = 6		
	Baseline	1 Month	∆	Baseline	1 Month	∆	Baseline	1 Month	∆
Weight (kg)	133.6	129.6	−4.0	116.6	114.8	−1.8	105.5 ± 2.4	102.5 ± 2.8	−2.9 ± 0.6
BMI (kg/m^2^)	45.2	44.1	−1.1	46.4	45.7	−0.7	44.9 ± 1.0	43.6 ± 1.1	−1.3 ± 0.2
Fat mass %	46.8	46.4	0.5	55.9	54.9	−1.0	53.5 ± 0.3	53.1 ± 0.4	−0.4 ± 0.2
Skeletal muscle mass %	30.0	30.4	0.5	24.5	25.0	0.5	25.8 ± 0.2	26.0 ± 0.2	0.2 ± 0.1
SBP (mmHg)	110	123	13	110	93	−17	111.6 ± 4.6	104.8 ± 4.7	−6.8 ± 4.8
DBP (mmHg)	80	86	6	75	66	−9	79.3 ± 3.2	73.5 ± 4.1	−5.8 ± 3.7
Glucose (mg/dL)	99.5	105.3	5.88	139.6	117.9	−21.7	105.2 ± 4.0	97.6 ± 2.4	−7.6 ± 4.8
Insulin (μU/mL)	16.9	20.8	3.87	30.7	32.9	2.18	25.2 ± 4.4	20.7 ± 3.3	−4.5 ± 6.1
HOMA-IR	4.16	5.4	1.25	10.6	9.6	−1.0	6.5 ± 1.1	4.9 ± 0.8	−1.5 ± 1.4
Adiponectin (μg/mL)	5.67	6.3	0.59	9.7	11.7	2.0	8.2 ± 1.8	7.6 ± 1.3	−0.6 ± 0.6
Leptin (ng/mL)	71.4	52.9	−18.5	98.7	92.5	−6.2	67.8 ± 6.2	57.3 ± 5.5	−10.5 ± 4.4

Data are shown as mean ± standard errors. MC4R, melanocortin 4-receptor; HOMA-IR, homeostatic model assessment insulin resistance; BMI, body mass index; SBP, Systolic blood pressure; DBP, Diastolic blood pressure.

**Table 3:** Comparison of anthropometric and biochemical parameters after 6 months of phentermine treatment (30 mg/day) in Ile269Asn carriers and non-carriers.

	Ile269Asn *MC4R* *n* = 6				Control Group *n* = 18				Difference between Groups	
	Baseline	6 Months	∆	*p*-Value	Baseline	6 Months	∆	*p*-Value	Mean Difference	*p*-Value
Weight (kg)	83.7 ± 4.3	70.9 ± 4.8	−12.7 ± 2.3	0.003	84.3 ± 1.6	72.9 ± 1.8	−11.3 ± 0.9	<0.001	−1.4 ± 2.1	0.523
BMI (kg/m^2^)	34.4 ± 1.3	29.2 ± 1.7	−5.2 ± 0.9	0.003	34.2 ± 0.4	29.6 ± 0.6	−4.5 ± 0.3	<0.001	−0.6 ± 0.8	0.447
% Fat mass	48.2 ± 2.4	43.6 ± 2.4	−4.6 ± 2.2	0.088	50.2 ± 0.6	45.4 ± 0.9	−4.8 ± 0.5	<0.001	0.1 ± 1.5	0.925
% Muscle mass	22.8 ± 1.3	24.0 ± 4.0	1.2 ± 0.9	0.242	21.5 ± 0.3	23.1 ± 0.4	1.5 ± 0.2	<0.001	−0.3 ± 0.6	0.630
SBP (mmHg)	105.0 ± 3.4	106.6 ± 2.4	2.0 ± 2.0	0.374	108.8 ± 2.2	103.3 ± 1.6	−5.5 ± 2.2	0.027	7.5 ± 4.3	0.100
DBP (mmHg)	75.0 ± 3.4	76.0 ± 2.4	2.0 ± 3.7	0.587	81.7 ± 5.4	72.2 ± 2.0	−9.5 ± 6.1	0.097	11.5 ± 12	0.264
Glucose (mg/dL)	97.3 ± 4.5	88.0 ± 3.5	−9.3 ± 4.9	0.129	94.2 ± 1.9	84.0 ± 1.7	−9.8 ± 2.4	0.001	0.4 ± 4.9	0.861
Insulin (μU/mL)	13.0 ± 2.9	8.2 ± 1.5	−4.8 ± 2.1	0.212	16.5 ± 2.2	8.3 ± 0.8	−8.4 ± 2.6	0.001	3.6 ± 4.4	0.328
HOMA-IR	3.2 ± 0.8	1.8 ± 0.3	−1.4 ± 0.6	0.179	3.9 ± 0.6	1.7 ± 0.1	−2.2 ± 0.7	<0.001	0.8 ± 1.2	0.365
Adiponectin (μg/mL)	3.7 ± 0.6	4.8 ± 0.7	1.1 ± 0.4	0.033	4.2 ± 0.4	7.0 ± 1.2	2.5 ± 1.2	0.062	−1.4 ± 2.0	0.900
Leptin (ng/mL)	24.7 ± 6.9	11.6 ± 3.4	−13.1 ± 7.5	0.067	20.2 ± 1.8	15.2 ± 3.3	−4.7 ± 4.1	0.039	−8.3 ± 8.0	0.484

Data are shown as mean ± standard error. Systolic and diastolic blood pressure, glucose, insulin, HOMA-IR, adiponectin, and leptin levels were log-transformed prior to the analysis. MC4R, melanocortin 4-receptor; BMI, body mass index; SBP, Systolic blood pressure; DBP, Diastolic blood pressure; HOMA-IR, homeostatic model assessment insulin resistance.

**Table 4:** Comparison of anthropometric and biochemical parameters 6 months after RYGB surgery in Ile269Asn carriers and non-carriers.

Parameter	Time	Ile269Asn *MC4R*	Control Group	*p*-Value
		*n* = 7	*n* = 24	
Age (years)	Basal	40.5 ± 2.9	40.9 ± 1.1	0.94
Weight (kg)	Basal	111.5 ± 3.6	110.9 ± 3.5	0.70
	6 months after RYGB	76.9 ± 4.7	79.4 ± 2.9	0.62
BMI (kg/m^2^)	Basal	43.8 ± 1.6	42.5 ± 1.3	0.70
	6 months after RYGB	30.0 ± 1.8	30.7 ± 0.9	0.48
%Hb1Ac	Basal	5.8 ± 0.3	5.7 ± 0.1	0.48
	6 months after RYGB	5.4 ± 1.2	5.3 ± 0.1	0.39
%WL	6 months after RYGB	29.9 ± 0.01	27.8 ± 0.01	0.73
%EWL	6 months after RYGB	66.6 ± 5.6	64.9 ± 3.1	0.51

Data are shown as mean ± standard error. BMI, body mass index; %Hb1Ac, glycosylated hemoglobin; %WL, weight loss percentage; %EWL, excess weight loss percentage.

## Data Availability

The original contributions presented in the study are included in the article/Supplementary Materials. Further inquiries can be directed to the corresponding author.

## Supplementary Materials

The following supporting information can be downloaded at: https://www.mdpi.com/article/10.3390/genes13122267/s1, Table S1. Comparison of basal anthropometric parameters in participants with obesity and normal weight; Table S2. Local ancestry percentage of a 1Mb segment containing the *MC4R* locus according to genotype; Table S3. Comparison of anthropometric and biochemical parameters after 3 months of phentermine treatment (30 mg/d) in Ile269Asn carriers and non-carriers; Table S4. Phentermine-related adverse events reported during the study in Ile269Asn carriers and non-carriers.
