# Supplementary material for: Effect of the Melanocortin 4-Receptor Ile269Asn Mutation on Weight Loss Response to Dietary, Phentermine and Bariatric Surgery Interventions
Source: Genes (Basel). 2022 Dec 1;13(12):2267. doi: 10.3390/genes13122267 (PMC9778600; doi:10.3390/genes13122267)
Supplement: Supplementary file 1 [file genes-13-02267-s001.zip › genes-2002524-supplementary.pdf]

Supplementary Materials

Supplementary Table S1. Comparison of basal anthropometric parameters in participants with obesity and normal-weight.

	**Obesity n=1200**	**Normal-weight n=483**	***P*-value**
Female; n (%)	1013 (84.4)	295 (61.1)	<0.001
Age	39.2±9.6	49.5±11.5	<0.001
BMI (Kg/m^2)	36.7±5.4	22.8±1.6	<0.001
**Ethnic admixture (%)**	**n=1062**	**n=482**	
Native American	64.0±15.3	56.4±21.4	<0.001
European	33.6±14.4	41.4±21.1	<0.001
African	2.3±2.3	2.2±2.5	0.065

Data are shown as mean ± standard deviation. *P*-values were calculated with U de Mann-Whitney non-parametric and chi-squared tests. BMI, body mass index.

Supplementary Table S2. Local ancestry percentage of a 1Mb segment containing the MC4R locus according to genotype.

		Ancestry proportion		
	**n**	**Native American**	**European**	**African**
Ile269Ile	198	58%	41%	1%
Ile269Asn	7	79%	21%	0%
Asn269Asn	1	100%	0%	0%

Supplementary Table S3. Comparison of anthropometric and biochemical parameters after 3 months of phentermine treatment (30mg/d) in Ile269Asn carriers and non-carriers.

	**Ile269Asn MC4R n=6**				**Control group n=18**				**Difference between groups**	
	**Baseline**	**3 months**	**Δ**	***P*-value**	**Baseline**	**3 months**	**Δ**	***P*-value**	**Mean difference**	***P*-Value**
Weight (kg)	83.7±4.3	74.5±3.9	-9.2±1.2	0.001	84.3±1.6	75.7±1.7	-8.6±0.7	<0.001	-0.5±1.5	0.713
BMI (kg/m^2)	34.4±1.3	30.7±1.3	-3.7±0.4	<0.001	34.2±0.4	30.7±0.5	-3.5±0.3	<0.001	-0.2±0.6	0.664
% Fat	48.2±2.4	46.2±22.2	-2.0±0.9	0.078	50.2±0.6	47.3±0.7	-2.9±0.5	<0.001	0.9±1.1	0.425
% Muscle	22.8±1.3	22.7±1.2	-0.03±0.3	0.917	21.5±0.3	22.2±0.3	0.6±0.3	0.067	-0.7±0.6	0.273
SBP (mmHg)	105.0±3.4	101.6±1.6	-3.3±3.3	0.405	108.8±2.2	104.4±1.8	-4.4±2.5	0.119	1.1±4.8	0.828
DBP (mmHg)	75.0±3.4	65.0±2.2	-10.0±4.4	0.086	81.7±5.4	73.3±1.8	-8.4±5.9	0.143	-1.5±10.6	0.632
Glucose (mg/dL)	97.3±4.5	91.8±4.2	-5.5±4.4	0.307	94.2±1.9	89.7±1.5	-4.8±2.0	0.029	-0.6±4.3	0.872
Insulin (µU/mL)	13.0±2.9	7.8±1.7	-5.2±1.9	0.143	16.5±2.2	9.4±1.2	-6.8±2.0	<0.001	1.6±3.6	0.958
HOMA-IR	3.2±0.8	1.8±0.4	-1.4±0.5	0.159	3.9±0.6	2.1±0.2	-1.7±0.5	<0.001	0.3±1.0	0.986
Adiponectin (µg/mL)	3.7±0.6	5.6±1.1	1.8±0.8	0.069	4.2±0.4	5.0±0.4	0.7±0.5	0.157	1.0±1.1	0.312
Leptin (ng/mL)	24.7±6.9	6.3±1.6	-18.3±7.4	0.018	20.2±1.8	18.9±3.2	-1.3±3.3	0.146	-17.0±7.1	0.012

Data are shown as mean ± standard errors. Systolic and diastolic blood pressure, glucose, insulin, Homa-IR, adiponectin, and leptin levels were log-transformed prior to analysis. MC4R, melanocortin 4-receptor; BMI, body mass index; HOMA-IR, homeostatic model assessment insulin resistance; SBP, Systolic blood pressure; DBP, Diastolic blood pressure.

Supplementary Table S4. Phentermine-related adverse events reported during the study in Ile269Asn carriers and non-carriers.

	**Ile269Asn n=6**			**Non-carriers n=18**		
	**Absence n (%)**	**Mild n (%)**	**Moderate n (%)**	**Absence n (%)**	**Mild n (%)**	**Moderate n (%)**
Dry mouth	1 (16.7)	5 (83.3)	0 (0)	0 (0)	18 (100)	0 (0)
Headache	2 (33.3)	3 (50.0)	1 (16.7)	3 (16.7)	5 (27.8)	10 (55.6)
Hyperhidrosis	4 (66.7)	2 (33.3)	0 (0)	7 (38.9)	11 (61.1)	0 (0)
Dysgeusia	4 (66.7)	2 (33.3)	0 (0)	8 (44.4)	10 (55.6)	0 (0)
Constipation	4 (66.7)	2 (33.3)	0 (0)	10 (55.6)	5 (27.8)	3 (16.7)
Nervousness	5 (83.3)	1 (16.7)	0 (0)	15 (83.3)	3 (16.7)	0 (0)
Insomnia	4 (66.7)	2 (33.3)	0 (0)	8 (44.4)	10 (55.6)	0 (0)
Drowsiness	6 (100)	0 (0)	0 (0)	16 (88.9)	2 (11.1)	0 (0)
Nausea	1 (16.7)	5 (83.3)	0 (0)	8 (44.4)	9 (50.0)	0 (0)
Anxiety	3 (50.0)	3 (50.0)	0 (0)	6 (33.3)	12 (66.7)	0 (0)
Fatigue	5 (83.3)	1 (16.7)	0 (0)	14 (77.8)	4 (22.2)	0 (0)
Irregular bowel sounds	5 (83.3)	1 (16.7)	0 (0)	16 (88.9)	2 (11.1)	0 (0)
Diarrhea	2 (33.3)	2 (33.3)	2 (33.3)	12 (66.7)	4 (22.2)	2 (11.1)
Abdominal pain	3 (50)	2 (33.3)	1 (16.7)	12 (66.7)	3 (16.7)	3 (16.7)
Dizziness	2 (33.3)	4 (66.7)	0 (0)	11 (61.1)	7 (38.9)	0 (0)

Data shown as number of patients with percentages in parenthesis.
